# Adult-Type Rhabdomyoma of the Larynx: Clinicopathologic Study of an Uncommon Tumor in a Rare Location

**DOI:** 10.1155/2017/7186768

**Published:** 2017-11-26

**Authors:** Giancarlo Altissimi, Massimo Ralli, Giulio Sementilli, Francesco Fiorentino, Andrea Ciofalo, Antonio Greco, Marco de Vincentiis, Alessandro Corsi, Giancarlo Cianfrone

**Affiliations:** ^1^Department of Sense Organs, Sapienza University of Rome, Rome, Italy; ^2^Department of Oral and Maxillofacial Sciences, Sapienza University of Rome, Rome, Italy; ^3^Department of Molecular Medicine, Sapienza University of Rome, Rome, Italy

## Abstract

Rhabdomyoma is an uncommon benign mesenchymal tumor with skeletal muscle differentiation that may occur either in the heart or in extracardiac sites. Even though the head and neck region is the most common area of extracardiac rhabdomyoma, the larynx is rarely involved. We present the case of an 85-year-old woman who reported a 10-day history of breathing difficulties, dysphagia, and dysphonia. A computed tomography scan of the head and neck showed a contrast-enhanced, solid hypopharyngeal-laryngeal neoplasm with well-defined margins causing subtotal obliteration of the right pyriform sinus and a reduction in air lumen of the laryngeal vestibule. The patient underwent complete endoscopic removal of the lesion; histologic examination revealed an adult-type rhabdomyoma based on the histologic features and the immunoreactivity of the neoplastic cells for desmin, myoglobin, and muscle-specific actin but not for cytokeratin, S-100, CD68R, chromogranin-A, and synaptophysin. Since clinical and imaging features are not specific for rhabdomyoma, histologic examination and immunohistochemical analyses play a central role in the differential diagnosis of the adult-type rhabdomyoma from other laryngeal neoplasms. A correct diagnosis is mandatory to avoid inappropriate treatment.

## 1. Introduction

Rhabdomyoma (RM) is an uncommon benign mesenchymal tumor showing skeletal muscle differentiation that may occur in cardiac and extracardiac locations [1]. Cardiac RM is considered a hamartomatous lesion that usually occurs in children with phakomatosis, such as tuberous sclerosis. In contrast, extracardiac RM is usually sporadic and may present at all ages. Depending on the degree of differentiation and the site of development, extracardiac RM is classified into foetal-, adult- and genital-type [1–3]. The foetal-type RM exhibits immature skeletal muscle differentiation and typically occurs as a solitary lesion in children. The adult-type RM exhibits mature skeletal muscle differentiation, occurs in adulthood with a median age of 60 years, and is usually, but not invariably, solitary. The genital-type RM shows an advanced degree of skeletal muscle differentiation and typically presents as a solitary lesion in the vagina, vulva, and cervix and, rarely, in the paratesticular region and epididymis of middle-aged patients. In terms of molecular genetics, both adult- and foetal-type RM have been associated with basal cell naevus syndrome and activation of the sonic hedgehog pathway [4, 5]. A t(15;17)(q24;p13) has been reported in one case of adult-type RM [6].

Even though the head and neck region is the most common area of extracardiac RM [1–3], this tumor is extremely rare in the larynx [7–38]. For this reason, we describe the clinicopathologic features of an adult-type RM of the larynx diagnosed in an 85-year-old woman and review the pertinent literature with the specific aim to assess the diagnostic and therapeutic standards for this uncommon tumor that are invariably necessary to avoid erroneous diagnosis and inappropriate treatment.

## 2. Case Presentation

An 85-year-old woman was referred to our institution with a 10-day history of breathing difficulties, dysphagia for solids and liquids, and dysphonia. The patient was a former smoker (20 cigarettes/day for 45 years until the age of 60), while no alcohol intake was reported. The patient's medical history was positive for chronic obstructive pulmonary disease, hypertension, anaemia, osteoporosis, goitre, and chronic renal failure.

**Figure 1:**
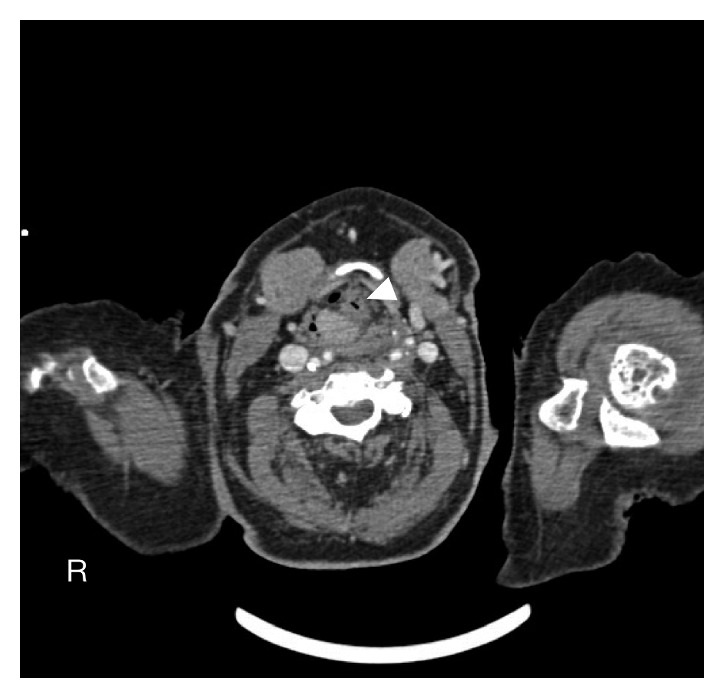
Contrast-enhanced computed tomography scan of the neck shows a large (18 × 22 × 12 mm) solid hypopharyngeal-laryngeal mass with well-defined margins, originating from the right pyriform sinus with partial contralateral extension (white arrow).

General otolaryngological examination was normal. Endoscopy revealed a submucosal lesion in the right arytenoid, covered with normal mucosa, extending towards the right pyriform sinus. The right vocal cord was fixed while the left was hypomobile, thus reducing breathing space. There was no clinical evidence of cervical lymphadenopathy. A computed tomography (CT) scan of the head and neck showed a contrast-enhanced, solid hypopharyngeal-laryngeal neoplasm with well-defined margins, originating from the right pyriform sinus with partial contralateral extension. The mass measured 18 × 22 × 12 mm and extended caudally to the right arytenoid and the upper margin of the cricoid cartilage, causing subtotal obliteration of the right pyriform sinus and a reduction in air lumen of the laryngeal vestibule. Other laryngeal structures were preserved (Figure 1).

The patient underwent complete endoscopic removal of the lesion and temporary tracheostomy for respiratory distress; tracheostomy was closed 32 days later. Neither postoperative complications nor signs of recurrence during an 18-month follow-up were observed.

**Figure 2:**
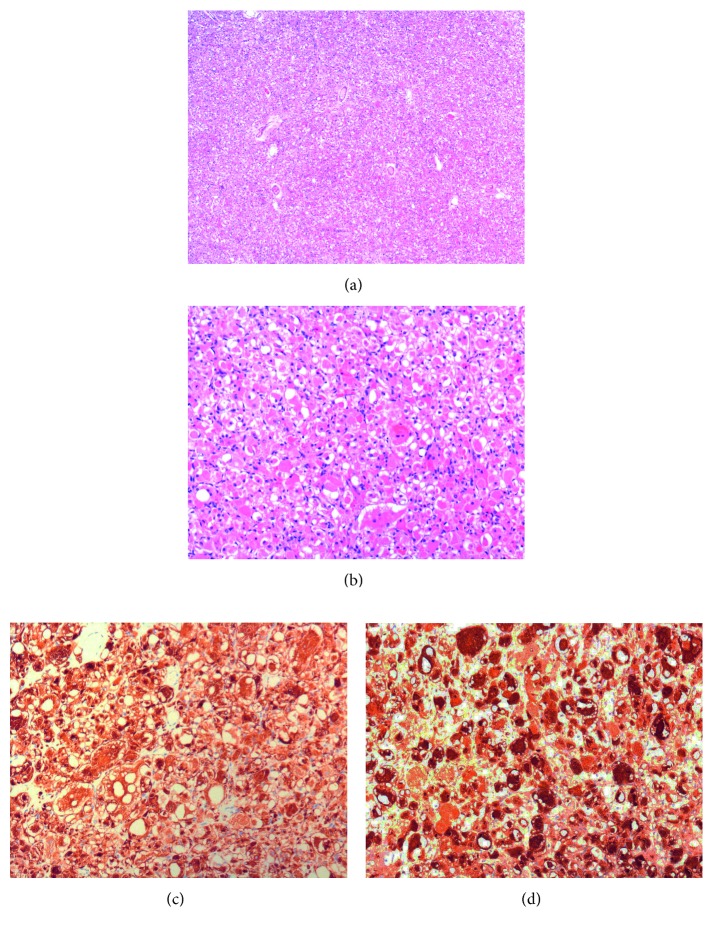
(a) Low- and (b) high-power magnification of tumor sections stained with haematoxylin and eosin. The neoplastic cells are diffusely positive for (c) desmin and (d) myoglobin.

Histologic examination of the neoplasm revealed a well-circumscribed, not encapsulated, proliferation of large closely packed neoplastic round to polygonal cells in a scant stroma (Figures 2(a) and 2(b)). The cells showed a deeply eosinophilic focally vacuolated cytoplasm and round, predominantly central, nuclei, at times with prominent nucleolus. In some cells, multiple nuclei were found. Cells with a peripheral cytoplasmic clear zone traversed by thin cytoplasmic strands extending from the more central acidophilic cytoplasm to the periphery (so-called “spider” cells) were detected as well. Mitotic activity and necrosis were absent. Neoplastic cells were immunoreactive for desmin (Figure 2(c)), myoglobin (Figure 2(d)), and muscle-specific actin. Immunohistochemical staining for cytokeratin (CKAEAE3 and CKMNF116), S-100 protein, CD68R, chromogranin-A, and synaptophysin failed to stain the neoplastic cells. Based on the histologic and immunohistochemical features, a diagnosis of adult-type RM of the larynx was made.

## 3. Discussion

About 90% of extracardiac RMs occur in the head and neck region [1–3,17]. Even though usually solitary, multifocal lesions have been described as well [27, 34]. The pharynx and oral cavity are the most commonly involved sites, while the larynx is an extremely rare location. Only twenty-three well-documented cases of RM of the larynx were reported in a review of the literature published in 1995 by Johansen; of these, 12 were adult-type laryngeal RM, 3 were adult-type multifocal RM, 4 were foetal cellular RM, and 4 were foetal myxoid RM [21]. Since then, no more than twenty other cases of adult-type RM have been published. A comprehensive list of adult-type laryngeal RMs reported in the literature is summarized in Table 1.

In the larynx, adult-type RM usually occurs as a solitary lesion in adulthood, is more frequent in men than in women, and more frequently involves the glottic and supraglottic regions [7–38]. In our case, the oldest patient in which an adult-type RM has been reported, the tumor involved primarily the arytenoid, an uncommon site of development for this neoplasm [18, 24, 28, 29, 31]. These tumors grow slowly as a submucosal painless swelling that progressively leads to hoarseness, foreign-body sensation, dysphagia, and dyspnea [3, 21]. Even though the duration of symptoms is usually long (typically years), airway obstruction and dyspnea may develop suddenly [15, 19]. In our case, the patient presented with a 10-day history of breathing difficulties, dysphagia for solids and liquids, and dysphonia. Imaging findings usually suggest a benign lesion because of submucosal location and absence of invasion of surrounding soft tissues. However, in particular on CT scan, adult-type RM may mimic malignant tumors because of indistinct borders blending into adjacent isodense muscles [24]. Thus, the absence of specific clinical and of unequivocal imaging features contributes to make any single case a diagnostic challenge. Preoperative differential diagnosis includes cysts, benign tumors (i.e., hemangioma, lipoma, neurofibroma, and granular cell tumor), and malignant neoplasms (i.e., squamous cell carcinoma and rhabdomyosarcoma).

Definitive diagnosis is possible only after histologic examination and immunohistochemical analysis. It is recommended to obtain the neoplastic tissue by excisional rather than incisional biopsy because in the latter the small amount of tumor tissue may contribute to misdiagnosis and inappropriate treatment [15, 37]. Independently of the site of the tumor, the histology of adult-type RM is quite distinctive [1]. As in our case, adult-type RM is composed by dense sheets of closely packed large, round cells with eosinophilic and granular cytoplasm, and centrally or peripherally located nuclei, in part with prominent nucleoli. Many cells are vacuolated because of intracellular glycogen that dissolves during processing. However, since these findings may resemble a variety of other lesions, in particular granular cell tumor, hibernoma, and paraganglioma, immunohistochemical staining is required for diagnosis. Neoplastic cells of adult-type RM—as reported in this case—are immunoreactive for desmin, myoglobin, and muscle-specific actin (markers of differentiated skeletal muscle cells) and negative for cytokeratin, S-100, CD68R, and neuroendocrine markers (synaptophysin and cromogranin-A).

The treatment of adult-type RM is surgical and can be done either by endoscopic or external approach, but always preserving the adjacent structures such as the vocal folds and the swallowing apparatus. Complete surgical excision is curative. Adult-type RMs may recur [3, 13, 18], especially after incomplete excision; however, aggressive behavior and malignant transformation have never been described.

In conclusion, the adult-type RM of the larynx is a very rare benign tumor that must be distinguished from other submucosal laryngeal neoplasms. The present case fulfills the clinicopathologic criteria for the diagnosis of adult-type RM of the larynx. Since clinical and imaging features are not specific for adult-type RM, histology and immunohistochemistry are mandatory for a definitive diagnosis. Radical endoscopic excision is the recommended procedure to obtain tissue for histologic evaluation and for patient treatment.

## Figures and Tables

**Table 1:** Clinical comparative synopsis of our case with the other cases of adult-type RM of the larynx reported in the literature, including the present case.

Ref.	Age/gender	Chief complaint	Treatment
	Location	Duration of symptoms	
[7]	48/M	Hoarseness	Endoscopic excision
	R vocal fold	3 months	

[8]	55/M	Hoarseness	Laryngofissure
	L transglottic area	3 years	Local excision

[9]	52/F	Hoarseness	Endoscopic excision
	R false vocal fold	3 years	

[11]	55/M	NA	Endoscopic excision
	R false vocal fold		

[12]	64/F	Hoarseness, foreign-body sensation	Endoscopic excision
	R ventricle	6 months	

[13]	39/M	Hoarseness	Endoscopic excision
	Vocal fold	3 years	

[14]	76/F	Hoarseness	Endoscopic excision
	L true vocal fold	2 months	

[15]	16/M	Acute airway obstruction	Tracheotomy
	R transglottic area	Sudden onset	Total laryngectomy

[17]	52/M	Hoarseness	Lateral pharyngotomy
	L vocal fold	6 months	

[17]	66/M	Hoarseness	Excision
	R vocal fold	8 years	

[18]	51/F	Dyspnoea, dysphagia	External removal
	Both artenoid cartilages	NA	

[21]	51/M	Hoarseness, snoring	Hemilaryngectomy
	L ventricular fold	1 year	

[22]	64/M	R asymptomatic submandibular mass	Cervical approach and L lateral pharyngotomia
	L aryepiglottic fold		

[23]	69/F	Hoarseness	Endoscopic excision
	Vocal fold	5 years	

[24]	39/M	Dysphagia and weight loss	Resection
	L-sided paraglottic space	5 months	

[25]	66/M	Hoarseness and dysphagia	External removal
	Arytenoid	4 months	

[26]	79/M	Hoarseness	External removal
	R false vocal fold	5 years	

[27]	69/M	R asymptomatic submandibular mass	External removal
	Epiglottis (multifocal)		

[28]	35/M	Cervical bulging	Resection
	Extrinsic laryngeal muscles	NA	

[29]	66/M	Dysphagia and hoarseness	Endoscopic excision
	R arytenoid	3 years (sudden dyspnea)	

[30]	72/F	Globulus and hoarseness	Endoscopic excision
	L aryepiglottic fold (multifocal)	1 year	

[31]	50/F	NA	Exeresis with CO_2_ laser
	R glossoepiglottic fold and vocal fold		

[32]	76/M	Hoarsness, dysphagia and sleep-apnoea	Multiple endoscopic multiple debulking procedures
	Arytenoid	NA	

[33]	NA/NA	Dysphonia	Endoscopic excision
	Glottis	NA	

[34]	55/M	Hoarseness and slight dysphagia	Endoscopic excision
	L paraglottic space (multifocal)	3 months	

[35]	35/M	Hoarseness	Endoscopic excision
	L supraglottic space	1 year	

[36]	67/F	Hoarseness and progressive dyspnea	Hemilaryngectomy
	Supraglottis	NA	

[37]	50/M	Change in voice	Total laryngectomy
	R aryepiglottic fold	2 years	

[38]	75/M	Hoarsness	Endoscopic excision
	R vocal fold	4 years	

Present case	85/F	Sudden breathing difficulties, dysphagia	Endoscopic excision
	R arytenoid	10 days	

R: right, L: left, NA: not available.
